# Empowering patient education on self-care activity among patients with colorectal cancer – a research protocol for a randomised trial

**DOI:** 10.1186/s12912-021-00617-z

**Published:** 2021-06-10

**Authors:** Leena Tuominen, Marita Ritmala-Castrén, Pia Nikander, Siru Mäkelä, Tero Vahlberg, Helena Leino-Kilpi

**Affiliations:** 1 Comprehensive Cancer Center, Helsinki University Hospital, Haartmaninkatu 4, 0029 Helsinki, Finland; 2 Department of Nursing Science, University of Turku|, Joukahaisenkatu 3-5, 20014 Turku, Finland; 3 Department of Nursing Management, Helsinki University Hospital, Stenbäckinkatu 9, 00029 Helsinki, Finland; 4 Department of Clinical Nutrition Therapy, Helsinki University Hospital, Tukholmankatu 8 F, 00029 Helsinki, Finland; 5 Medical Faculty, University of Helsinki, Haartmaninkatu 8, 00029 Helsinki, Finland; 6 Department of Clinical Medicine, Biostatistics, University of Turku, Kiinamyllynkatu 10, 20520 Turku, Finland; 7 Turku University Hospital, Kiinamyllynkatu 4-8, 20521 Turku, Finland

**Keywords:** Empowerment, Education, Self-care, Chemotherapy, Nutrition, Side effect, Patients with colorectal cancer

## Abstract

**Background:**

Chemotherapy-induced side effects may have a negative effect on nutrition intake, thus increasing the risk of malnutrition and consequently, other serious complications for patients with cancer. The prevalence of malnutrition is common among patients with colorectal cancer. Nurse-led empowering education may have a positive effect on self-care activity in this patient group. Therefore, our purpose is to develop an empowering educational nursing intervention and test its effect on self-care activation and knowledge level among patients with colorectal cancer during chemotherapy. Secondary outcomes are quality of life and risk of malnutrition.

**Methods:**

An interdisciplinary expert group developed a face-to-face empowering educational intervention using teach-back method. A two-arm, single-centre, superiority trial with stratified randomisation (1:1) and pre-post measures will be used to assess the effect of the intervention compared to standard care. Patients (*N* = 40 + 40) will be recruited in one university hospital outpatient clinic in Finland. Eligibility criteria are adult patients diagnosed with colorectal cancer starting oral fluoropyrimidine or combination chemotherapy treatment. A registered nurse experienced in oncology will deliver the intervention 2 weeks after the first chemotherapy. Outcomes are measured before intervention (M0) and after a two-month follow-up period (M1).

**Discussion:**

This study will assess whether nurse-led empowering education using teach-back method is effective on self-care activity among patients with colorectal cancer. If the intervention has a positive effect, it may be implemented into patient education in a corresponding context.

**Trial registration:**

ClinicalTrials.gov: NCT04160650 Registered 12 November 2019 - retrospectively registered

## Background

People are increasingly affected with colorectal cancer (CRC), which is one of the most prevalent cancers globally, comprising about 10% of newly found cases [1, 2]. In Finland, about 3300 patients are diagnosed annually with CRC, making it the second common cancer type among both men and women [3]. Chemotherapy is a common treatment for patients with operated high-risk stage II and III CRC as well as advanced and metastatic CRC [4]. Chemotherapy-related toxicities that affect the ability to eat are known as nutrition impact side effects (NIS). Side effects such as nausea, diarrhoea, constipation, mouth sores, heartburn, loss of appetite, altered taste, cold sensitivity, pain, fatigue and distress may lead to inadequate nutritional intake and weight loss, thus increasing the risk of malnutrition [5–8].

Malnutrition is common among patients with CRC, the prevalence varying according to patients’ age, cancer type and stage of cancer [7, 8]. In malnutrition, a deficiency or excess of energy, protein, and other nutrients causes measurable adverse effects on body function and clinical outcome [9]. The prevalence of both malnutrition and NIS are higher in older population [10]. For example, among geriatric patients with gastrointestinal system cancer (*n* = 153) about 38% were malnourished and 35% at risk of malnutrition at the time of the first outpatient visit. Chemotherapy has been shown to increase the incidence of malnutrition and weight loss [7, 11]. The complications of severe malnutrition may lead to greater chemotherapy toxicity, worse physical function and quality of life (QoL), and reduced overall survival [12, 13]. Moreover, malnourished inpatients have longer hospital length of stay and higher treatment costs [8]. Therefore, it is essential to develop effective interventions to support empowerment and promote nutrition intake of patients receiving chemotherapy.

Nutrition-related interventions for patients with cancer have been studied extensively with mixed results. Interventions have included individualised nutritional support and counselling to reach protein and energy goals [14], personalised nutrition intervention [15–17], dietary counselling or advice [18, 19], oral nutritional supplements (ONS), and nutrition advice with written information [18]. Interventions have proved to be effective on energy and protein intake [14–19], weight [15], QoL [14, 16, 17, 19], morbidity and mortality [14, 16, 17] as well as the risk of adverse clinical outcomes at 30 days [14]. Among patients with head and neck cancer undergoing radiotherapy, individualised nutritional counselling compared to ad libitum diet and ONS was capable of sustaining a significant impact on patients’ outcomes after 3 months’ follow-up [16]. Conversely, some of the interventions mentioned above have not been effective on weight [18, 19], nutritional status, QoL, functional status [18] and mortality. According to authors, the lack of effect might have been related to small sample sizes, short follow-up periods or low methodological quality of the studies [19]. Alternatively, Internet-based interventions including information and some interactive activities with experts to manage common eating and nutritional problems during cancer treatments have been tested. The results have not shown statistically significant changes on patients’ knowledge levels, anxiety and QoL, probably due to limited sample size or insufficient intervention [20].

Only few studies have explored the effect of nurse-led nutritional interventions among this patient group, yet the results have been promising. A multidisciplinary team approach for nutritional interventions (individual recipes, nutritional risk screening, total energy requirement calculation, education and diet adjustments) conducted by specialist nurses has obtained a positive effect on pre-albumin levels among CRC patients undergoing chemotherapy [21]. An individualised educational program with face-to-face and telephone counselling gained positive results on energy and total protein intake among patients with CRC (*n* = 19 + 21) in palliative care context [22]. The same type of intervention among patients with gastric cancer (*n* = 72 + 72) implemented by a nurse specialist had a positive effect on nutritional intake, haemoglobin, total serum and albumin levels, as well as on chemotherapy compliance rate [23]. Our literature search on the subject did not find other results of nurse-led studies, so there is still a lack of nursing-specific outcomes of nutrition-related interventions. In general, educational nursing interventions have shown positive outcomes on the level of knowledge and symptom severity yet the results have been inconsistent on QoL among patients with cancer [24].

Self-care at home between the chemotherapy cycles has an important role in the success of overall care. Self-care involves both the ability to care for oneself and the activities necessary to achieve, maintain, or promote one’s optimal health. Through self-care, various outcomes may be achieved; for example, improved symptom control, coping with the illness, and QoL. In addition, health services usage and costs may decrease [25]. In this study, self-care is seen as an ability to manage NIS and gain control over one’s health. We use empowering patient education and teach-back method to support patients in their self-care.

Empowerment refers to the ability to manage the challenges of the illness and having a feeling of control over one’s life. It is perceived as an inner strength of a human [26, 27]. Empowerment occurs when individuals’ capacity to think critically and make informed decisions is supported and they make decisions about their own care [28]. Empowerment is created in dialogue in nurse-patient relationship [29] as nurses support patients by offering knowledge and assist them to find, construct and use their own resources in self-care. The dimensions of empowerment can be categorised as experiential (patients’ earlier experiences), functional (function of one’s body and mind), ethical (feeling of being valued and respected), financial (affording support, technical aids), bio physiological (knowing one’s own body and its symptoms), social (interaction with other people) or cognitive (knowledge for improving one’s health) [26, 30]. In this study, empowerment is seen as a process where the nurse supports patients’ empowerment to be more active in self-care. During this process, patients gain knowledge to develop skills for NIS-related problem-solving in daily life [27, 28] in order to reduce the risk of malnutrition and promote the QoL.

Previous studies examining patients’ expectations towards their care demonstrate the expectation of having knowledge [31–33] to manage NIS and own health independently at home. As nutritional care is seen as part of fundamental care [34], nurses have a good opportunity to support patients’ empowerment on self-care of NIS and prevent the risk of malnutrition during chemotherapy. With this research protocol, we answer the question how the empowering educational nursing intervention using teach-back method will be tested on self-care activation, knowledge level (primary outcomes), QoL and risk of malnutrition (secondary outcomes) among patients with CRC during chemotherapy.

## Methods

### Aim, design and setting of the study

**Fig. 1:**
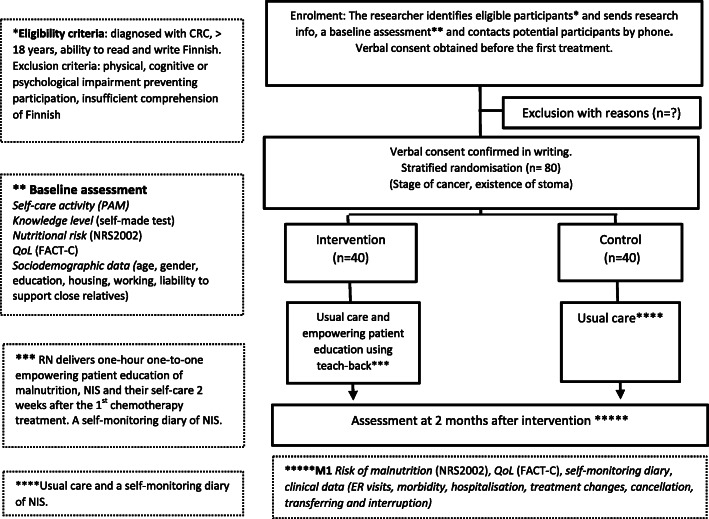
Study design

The aim of this intervention is to improve patients’ empowerment in self-care. The design is a two-arm, single-centre trial with stratified randomisation (1:1) with repeated measures (Fig. 1). We hypothesise that patients with CRC who receive nurse-led empowering education of NIS vs standard education have higher self-care activation level, better knowledge level, less risk of malnutrition and less worsening of QoL at 2 months’ follow-up compared to the control group (CG).

The study setting is a large university hospital in Southern Finland, responsible for specialised health care for about 2.1 million people. In the Cancer Centre outpatient clinic, about 4500 patients diagnosed with CRC (colon, rectum) receive chemotherapy treatment annually. Up to 40 new patients per month come for an evaluation of chemotherapy initiation.

### Characteristics of participants

Eligible participants are adult men and women diagnosed with CRC, > 18 years and having oral fluoropyrimidine or combination chemotherapy treatment. Exclusion criteria are physical, cognitive or psychological impairment preventing participation and insufficient comprehension of Finnish language.

### Intervention

#### Standard care

Patients both in CG and intervention group (IG) receive standard education delivered by a registered nurse (RN) during the first visit in the outpatient clinic and later on in the infusion unit. Standard education includes the following verbal information:

- side effects and their self-care; nausea, diarrhoea, obstipation and sores in the mouth, peripheral neuropathy symptoms, local venous irritation, heart symptoms, mucous and skin irritation
- self-monitoring of NIS, fluid intake, medication dose changes, effect of chemotherapy
- weight control
- taste alteration
- cold sensitivity
- importance of varied diet
- oral nutritional supplements
- clinical nutritionist services

Standard education includes the following written information:

- ‘Nutrition guide for cancer patients’
- ‘Instructions for those receiving anticancer treatment’
- ‘Information on strong opioids’
- ‘Cancer pain management’
- ‘Anti-nausea medication’
- ‘When you have nausea’
- ‘Management of diarrhoea’
- ‘Management of constipation’
- ‘Oral care instructions for cancer patients’

In addition to a RN, physicians give patients instructions on medication and related side effects.

#### Intervention protocol

The intervention was developed during autumn 2018 and spring 2019 by an interdisciplinary expert group consisting of two RN experienced in oncology, a clinical nutritionist, an oncologist, and the researcher (LT). We held four shared meetings and additional discussions between each member and the researcher. According to the protocol, patients in IG will receive knowledge of healthy diet and malnutrition. In addition, they will receive tailored knowledge of NIS. The tailoring is based on the side effect self-monitoring diary and patient activation level assessment according to the Patient Activation Measure (PAM) [35]. In addition, patients receive knowledge of the prevention of NIS and self-care strategies based on organisation guidelines. The teach-back method is used to verify participants’ understanding of the received knowledge, to tailor education, to uncover health beliefs, and to activate patients in dialogue [36]. Participants have to be able to teach back the main parts of the knowledge related to malnutrition as well as the reasons, prevalence, and self-care of the NIS they are suffering from. The teach-back method has shown a positive effect in self-care by improving outcomes in disease-specific knowledge, adherence and self-efficacy among people with chronic disease. It has also reduced hospital readmission rates [37].

Empowerment is supported by offering additional knowledge according to patients’ expectations. In addition, the research nurse uses active listening and strengthening self-care strategies that have been successful. The progression of the discourse is based on patients’ expectations and active involvement: the nurse provides the patient with expert knowledge and maintains an empathic connection to the patient throughout the session [38].

The content of the educational intervention is as follows:

- Illustrating the purpose of the session.
- Exploring patients’ current knowledge of healthy diet and malnutrition, offering additional knowledge using teach-back.
- Bringing forward individual NIS and their intensity on a numerical rating scale (NRS) ≥3, and patients’ knowledge expectations, offering additional knowledge using teach-back.
- Bringing forward performed self-care strategies and offering additional knowledge using teach-back.
- Bringing forward individual NIS and their intensity (NRS < 3), offering additional knowledge.
- Making a brief summary.

Due to the COVID-19 pandemic, the intervention was scheduled for the second chemotherapy course. The intervention will not entail any extra costs for patients. In case the intervention cannot be conducted face-to-face, it is possible to make it available online, e.g. as a video call.

#### Intervention nurse

The eligibility criteria for the research nurse comprise being a registered nurse and experienced in oncology nursing. The research nurse is trained to deliver the intervention by the researcher during three-hour face-to-face sessions held three times. The research nurse answers open-ended questions related to the content and method of education before the intervention commences. The intervention is delivered systematically according to the prescheduled protocol. After each session, the research nurse documents the length and content of the intervention. The researcher and research nurse will meet once a week to check that the protocol is adhered to.

### Outcome measures

**Fig. 2:**
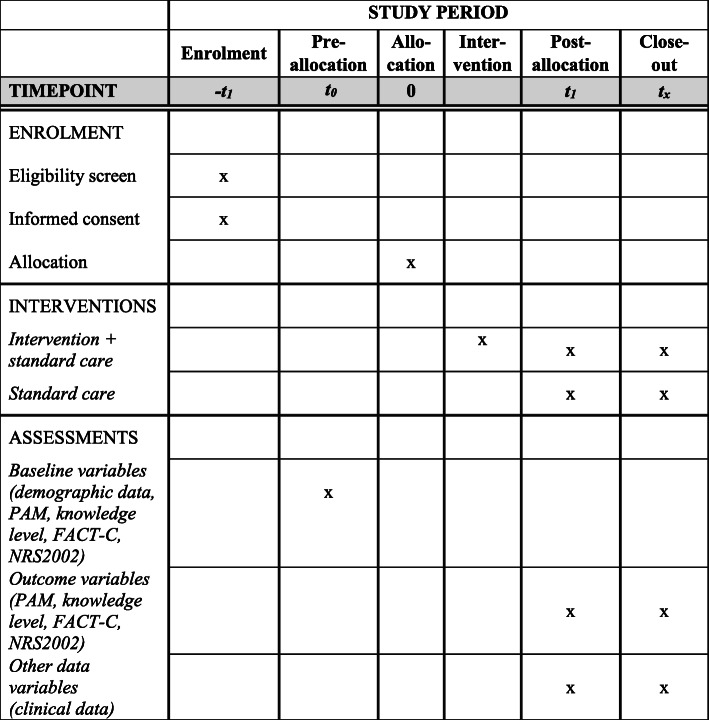
Schedule of enrolment, interventions, and assessments

Primary outcomes are activation in self-care and knowledge level. Secondary outcomes are risk of malnutrition and QoL. The schedule of enrolment, interventions, and assessments [39] is presented in Fig. 2.

#### Self-care activation

Self-care activation is measured, as achieving self-management is one of the most frequent consequences associated with patient empowerment [29]. In addition, there is evidence that patient activation is associated with health-related outcomes. It has been found that active people are more likely to have received preventive care, less likely to smoke or have a high BMI, and have better clinical indicators (systolic blood pressure, LDL). In addition, they are less likely to be hospitalised or use emergency services [40]. Self-care activation is measured using the Patient Activation Measure (PAM) instrument [41], which measures patient activation, a concept related to empowerment. The instrument covers the elements used to define empowerment (patients’ capacities, knowledge, behaviours and support by others) [42]. PAM measures individuals’ knowledge, skills and confidence to manage their own health. The questionnaire consists of 15 items on a 5-point Likert scale. Individuals fall into one of the following levels of activation along a 0–100-point scale: 1) Being overwhelmed and unprepared to play an active role in own health, 2) lacking knowledge and confidence for self-care, 3) taking action but lacking confidence and skill to support behaviours, 4) adopting health supporting behaviours, but may have difficulties to maintain them in stressing situations. The measure has proved to be highly valid and reliable with good psychometric properties, indicating its use in tailoring interventions and assessing changes. In this study, PAM scores are used to support patient activation individually. Creating situations where patients can experience success in taking control of their health is an essential part of effective self-care support [41, 43, 44].

#### Knowledge level

Knowledge is seen as essential for empowerment [29]. Knowledge tests have been used to measure the outcomes in educational interventions, to evaluate their effect or to monitor the learning progress during education [45]. Positive outcomes have been reported on disease-specific knowledge, adherence to medication and diet as well as on self-efficacy among people with chronic disease. A positive but inconsistent effect has also been reported in self-care and hospital readmission rates [37]. For this study, a knowledge test based on literature was developed in the research group consisting of a clinical nutritionist, a physician, nurses experienced on oncology and the researcher. A clinical nutritionist and two patient experts validated the test. The responses required (15 items) are either yes or no. Each correct answer gives one point and total score is the sum of correct answers. The knowledge test covers the following topics:

1. Malnutrition; definition and prevalence in patients with CRC (2 items)
2. Impact of malnutrition on treatment, morbidity and mortality (2 items)
3. Chemotherapy-induced side effects that may reduce nutritional status; reasons, manifestation and self-care (11 items)

#### Quality of life

QoL is measured as the risk of malnutrition is strongly associated with QoL in cancer patients initiating adjuvant chemotherapy [46]. Improved QoL is seen as a long-term consequence of patient empowerment [29]. QoL is assessed using the Functional Assessment of Cancer Therapy Scale – Colorectal (FACT-C) [47], which is a reliable and valid measure and sensitive to changes in functional status [48]. The questionnaire consists of 36 items on a 5-point Likert scale in four areas of wellbeing: physical (0–28 points), social (0–28 points), emotional (0–24 points), functional (0–28 points) and CRC subscale (0–28 points). Total sum is 0–136 points. Higher score means better QoL. Points are assigned for low level (0–34 points), satisfactory (34–68 points), average (68–102 points) and high level (102–136 points). FACT-C has been shown to have good overall validity and reliability, to be short, have flexible scoring, responsiveness to change in performance status, to be significantly correlated with other assessments of mood and show positive results in hypothesis testing [49, 50].

#### Risk of malnutrition

The risk of malnutrition is assessed as the educational intervention is presumed to prevent malnutrition or reduce the risk of malnutrition. In diagnostic assessment of malnutrition, the following criteria are recommended: non-volitional weight loss, low body mass index (BMI), reduced muscle mass, reduced food intake or assimilation and disease burden/inflammation [51]. To identify the patients at risk, we use the validated Nutritional Risk Screening 2002 tool (NRS2002) [52], which was developed to detect the presence of malnutrition and to predict whether malnutrition is likely to worsen due to patients’ illness [53]. Patients are assessed based on BMI, recent weight loss percentage and change in food intake (0–3 points), age (0–1 points) and severity of disease (0–3 points). The total sum is seven points. Patients with a total score of ≥3 are classified as nutritionally at risk.

#### Side effects

Side effect self-monitoring is used to assess the intensity of the side effects as well as to reinforce the intervention effect by reflecting on the self-care activities. After the first chemotherapy treatment, nurses give the self-monitoring diary to patients in both IG and CG, to be returned to the researcher after the fourth (or the last) chemotherapy cycle. Patients in IG document the side effects and their intensity as they appear (NRS 0–10; 0 = not at all, 10 = the worst possible) before and after the performed self-care activities. In addition, patients document their individual expectations for additional knowledge, and this information is used during the educational intervention. Patients in CG only self-monitor and document the intensity of each side effect as it appears (NRS 0–10; 0 = not at all, 10 = the worst possible).

#### Clinical data

Clinical data is gathered from electronic patients’ records. We are interested to find out whether the educational intervention is related to better adherence to treatment schedule. Therefore, data of patient-induced treatment changes, cancellations, transferring and interruptions are documented from baseline to 8 weeks. It has been indicated that worse nutritional status is related to greater morbidity [17]. Therefore, we collect data of patients’ emergency room visits and hospitalisation from baseline to 8 weeks.

The sample size was calculated to detect a 7-point mean difference in the PAM scale between groups assuming standard deviation of 11 points for both groups with 80% power and significance level of 0.05. This leads to required sample size of 40 participants per group. The meaningful difference between the average score of individuals who engage in healthy behaviours and those who do not is considered 4 points on the PAM scale [44]. To reach the target sample size (40 + 40), the researcher recruits participants by sending questionnaires with a research info and contacting them by phone the day before they visit the outpatient clinic. The researcher also meets the eligible participants to provide verbal information of the trial and answer questions. The strategies to improve patients’ adherence to the intervention protocol comprise the use of a self-monitoring diary and positive feedback from the research nurse. For individual participants, the intervention will be discontinued in the case of worsening condition, treatment change, or at their own request.

### Assignment of interventions

The researcher (LT) enrols the participants, and those willing to participate are randomly assigned to CG and IG using stratified randomisation according to stage of disease and existence of stoma. An allocation sequence using blockrand package [54] in R version 3.6.1 [55] was used. For each block, the block size was randomly chosen from a set of 2, 4 and 6. Allocation ratio of 1:1 was used. The statistician generated an unpredictable allocation sequence using sequentially numbered, opaque, sealed envelopes. The envelopes, numbered in advance, are opened sequentially after the participant’s name is written on the appropriate envelope [56]. The researcher (LT) allocates participants to the intervention with equal probability as a simple random sample is drawn from each group. Thus, the person enrolling participants does not know in advance which treatment the next person will get [39]. Patients' blinding is not possible as the RN informs the patients in IG of the one-hour educational session. The research nurse cannot be blinded because she provides the intervention. The data analyst is blinded as data are anonymised by using codes (001, 002 etc.).

### Data collection

Baseline measurement (M0) is conducted before patients’ first contact in the outpatient clinic. Follow-up measurement (M1) is conducted 8 weeks after the intervention. The duration of a chemotherapy treatment varies from 3 to 6 months. The occurrence of chemotherapy side effects is individual and they are principally temporal being most severe in 3–7 days after each cycle. During the two-month follow-up period, patients have received four cycles of treatment, which is considered sufficient to assess the effects of the intervention. The researcher sends research information and baseline questionnaires (demographic data, PAM, knowledge test, FACT-C, NRS2002) to the patients with a return envelope enclosed. She contacts potential participants by phone before their first treatment to inform about the study and asks for their verbal consent. Patients return the questionnaires within a week before the first appointment in the outpatient clinic. In connection with this appointment, the researcher contacts the participants for verbal research info and written informed consent. Recruitment continues until the sample size is reached (40 + 40). The enrolment period will last 4–5 months based on calculated sample size and the assumption of 10 new CRC patients a week and that approximately 10% of the patients will refuse to participate. Outcome data is not collected on participants who suspend on their own initiative or deviate from intervention protocols. The researcher manages data confidentially by entering and storing data on a password-protected computer. Original study questionnaires are kept at the participating site. The researcher codes data for ease of data storage, review, tabulation, and analysis. Files in electronic and paper form will be discarded after the publication of the research results (2022). In the information letter, patients are instructed to report spontaneously any unintended effects to an outpatient clinic nurse. Nurses report these events to the physician and first author, who make the final decision to discontinue the study, if necessary. A formal data monitoring committee will not be set up due to the short duration of the study and the harms are known to be minimal.

### Data analysis

Statistical methods are used for analysing primary and secondary outcomes between the two groups and related factors. The main analysis is to compare the change from M0 to M1 in primary outcomes (patient activation and knowledge level) and secondary outcomes (QoL and risk of malnutrition) between IG and CG. Categorical variables will be described using frequencies and percentages. Continuous variables will be expressed as means with standard deviations for normally distributed variables and medians with interquartile ranges for non-normally distributed variables. The differences in changes between groups in continuous outcomes will be compared with two-sample t-test or Mann-Whitney U-test and the changes within groups with paired t-test or Wilcoxon signed rank test, as suitable. Logistic regression using generalised estimating equation (GEE) will be used to test the differences between and within groups at risk of malnutrition (classified as 0–2 and 3–7). Results will be presented using estimates of group differences with 95% confidence intervals. The effect of missing data on the results will be examined using sensitivity analysis. An intent-to-treat analysis will be applied and two-sided statistical tests with significance level of 0.05 will be used in the statistical analyses. Methods for any additional analyses are determined by the data.

### Declaration of interests

None.

### Access to data

The searcher has access to the final trial dataset. A contractual agreement is made with the statistician to handle the data confidentially according to the research protocol.

## Discussion

We developed a study protocol to test the effectiveness of an empowering educational nursing intervention using the teach-back method on the self-care activation and knowledge level (primary outcomes), QoL and risk of malnutrition (secondary outcomes) among patients with CRC during chemotherapy. This study is currently in the recruitment phase (first enrolment 21.10.2019). Today, various Internet-based interventions are offered for patients with cancer. A vast amount of information is available from different sources. Patients with colorectal cancer are usually older people and the Internet is not available for all. Individualised interventions have proved effective on patients’ energy and protein intake, weight, QoL, morbidity, mortality and the risk of adverse clinical outcomes. Previous studies have concentrated merely on clinical outcomes and QoL. Therefore, we are interested to test the effectiveness of face-to-face individualised education on patients’ self-care activation and knowledge level. The intervention has potential to improve nutritional outcomes for patients affected with CRC. If the empowering patient education with teach-back method proves to be effective, it will be implemented as a part of RNs’ daily work. Further research will provide valuable information of costs and benefits when implementing this educational programme. At the protocol phase, the cost of training the intervention nurse and staff (20 h) and the salary cost of the research nurse (8 months) is approximately € 15,000.

## Data Availability

Not applicable.

## Abbreviations

CRC: Colorectal cancer
QoL: Quality of life
NIS: Nutrition impact side effects
ONS: Oral nutritional supplements
CG: Control group
\IG: Intervention group
RN: Registered nurse
NRS: Numerical rating scale
BMI: Body mass index
GEE: Generalised estimating equation
HUH: The Helsinki university hospital
PAM: The patient activation measure
FACT-C: The functional assessment of cancer therapy scale – colorectal
NRS2002: The nutritional risk screening 2002 tool
